# Behavioral tagging and capture: long-term memory decline in middle-aged rats

**DOI:** 10.1016/j.neurobiolaging.2018.02.023

**Published:** 2018-07

**Authors:** Alexandra Gros, Szu-Han Wang

**Affiliations:** a Centre for Clinical Brain Sciences, The University of Edinburgh, Edinburgh, Scotland, UK; b Centre for Cognitive Ageing and Cognitive Epidemiology, The University of Edinburgh, Edinburgh, Scotland, UK

**Keywords:** Memory consolidation, Reconsolidation, Memory modulation, Normal aging, Reward learning

## Abstract

Decline in cognitive functions, including hippocampus-dependent spatial memory, is commonly observed at a later stage of aging (e.g., >20 months old in rodents) and typically studied after a discrete learning event. How normal aging, particularly at an early stage, affects the modulatory aspect of memory persistence is underinvestigated. Previous studies in young animals show that weak, fading memories can last longer if a modulating event, such as spatial novelty, is introduced around memory encoding. This is known as behavioral tagging and capture (BTC). Here, we investigated how early aging (10–13 months old) affects BTC in an appetitive delayed-matching-to-place task. We trained rats when they were young and middle aged and found that novelty facilitated long-term memory persistence in young but not in middle-aged rats. However, re-exposure to the encoded environment after learning improved memory persistence in middle-aged rats. BTC, combined with memory reactivation, facilitated memory persistence through reconsolidation. Our results point toward a weakened tagging and capture mechanism before reduction of plasticity-related proteins at an early stage of aging.

## 1 Introduction

Aging is a natural biological process associated with a decline in cognitive function (Leal and Yassa, 2015). Age-related impairment in spatial (Bohbot et al., 2012, Techentin et al., 2014) and episodic memory (Isingrini and Taconnat, 2008, Rönnlund et al., 2005, Spencer and Raz, 1995) is common in humans. Similarly, deficits in navigational strategy, spatial memory, pattern separation, and reductions in working memory capacity are also observed during normal aging in animals (Bach et al., 1999, Barnes, 1979, Creer et al., 2010, Cès et al., 2018, Dunnett et al., 1988, Lacreuse et al., 2014, Markowska et al., 1989, Rapp et al., 1997). Memory decline has often been documented at a later stage of aging (e.g., >20 months old in rodents), but results obtained in the middle age (10–16 months old) show mixed findings (Aitken and Meaney, 1989, Das and Magnusson, 2008, Fouquet et al., 2011, Frick et al., 1995, Magnusson et al., 2003, Stouffer and Yoder, 2011, Verbitsky et al., 2004), and how memory is affected at midlife remains relatively understudied.

Memory persistence reflects a highly dynamic process that involves memory encoding, modulation, consolidation, and reconsolidation (Dudai, 2012, Wang and Morris, 2010). Understanding which one of these processes is the first to be impacted by aging can provide valuable information on how to improve cognitive wellbeing during healthy aging. To describe the mechanisms affected by the early phase of aging, we used a behavioral tagging and capture paradigm. This provides a method for dissociating the process of encoding the memory of interest and the facilitation of memory persistence by a memory-modulating event (MME).

Behavioral tagging and capture (Ballarini et al., 2009, Moncada et al., 2015, Wang et al., 2010) refers to the process of facilitating memory persistence that follows the same principle of facilitating the persistence of plasticity change seen in synaptic tagging and capture (STC). Specifically, in synaptic plasticity assays in the CA1 region of the hippocampus, a normally decaying early phase of long-term potentiation (LTP), induced by weak tetanus stimulation, can persist at late-phase if strong stimulation is applied in a separate but converging pathway within a critical time window around the weak stimulation. This facilitation of LTP persistence does not occur if protein synthesis is inhibited during the strong stimulation (Frey and Morris, 1997). It is hypothesized that a weak tetanus alone leads to production of tags at stimulated synapses, which are insufficient to support late-phase LTP. In contrast, a strong tetanus leads to production of tags at stimulated synapses and plasticity-related proteins or products (PRPs) in the stimulated cells, which contribute to late-phase LTP. If a weakly stimulated pathway and a strongly stimulated pathway engage overlapping neuronal populations, and the strong tetanus is applied at about 30–60 minutes before or after the weak tetanus, PRPs triggered by the strong tetanus can be captured by the tagged synapses and lead to late-phase LTP in both pathways (Frey and Morris, 1998, Sajikumar and Frey, 2004). This “strong-converting-weak” effect, normally observed in in vitro hippocampal preparations, has also been shown in in vivo recording (Shires et al., 2012). STC provides a potential cellular mechanism of long-term plasticity changes, and its underlying molecular correlates have been widely studied (for review, see Redondo and Morris, 2011). Following the “strong-converting-weak” effect seen in electrophysiology, a similar phenomenon has been observed in long-term memory at the behavioral level. When a weak learning event, usually leading to short-lasting memory, is followed by or preceded with a strong event, the memory can become long lasting. For example, exploration in a novel open field can facilitate the persistence of aversive types of memory such as inhibitory avoidance and contextual fear memory (Ballarini et al., 2009, Moncada and Viola, 2007).

Applying this facilitation effect to appetitive memories, we have developed an appetitive spatial memory task for rats (Bast et al., 2005, Wang et al., 2010) that is similar to the everyday experience of humans when remembering where events are hosted or where cars are parked. This task involves training the animal to locate where a reward is hidden and later find more rewards in the matching location among other nonrewarded locations. We observe that weak encoding, that typically leads to fading memory within a day, can persist for 1 day if the initial encoding trial was followed by exploration in a novel context (Wang et al., 2010). How aging affects the persistence of appetitive spatial memory (i.e., memory of interest), and what kind of MME facilitates memory persistence can be addressed using this paradigm. We hypothesized that changing the encoding strength (strong or weak), the delay before the memory tests, and the type of MMEs would reveal whether it is the process of tagging and capture or the process of producing PRPs, which is primarily impaired during aging.

To this end, we trained a cohort of rats to carry out this appetitive delayed-matching-to-place task when they were young and then when they were middle aged. We found that novelty facilitated the long-term persistence of memory after weak encoding in young but not in middle-aged rats. Young rats showed long-lasting memory after strong encoding, whereas middle-aged rats did not, and the memory in the middle age was not facilitated by novelty. Importantly, the memory persisted longer in middle-aged rats if an opportunity for re-encoding, through nonrewarded re-exposure or a rewarded second trial, was provided. Finally, when novelty was introduced after memory reactivation, it enabled memory persistence through reconsolidation. Our results suggest that the behavioral tagging and capture process is deteriorating during cognitive aging, which contributes to poor memory persistence.

## 2 Materials and methods

### 2.1 Animals

Adult male Lister Hooded rats (Charles River, 200–225g on arrival, n = 16) were group housed (4 per cage) in a temperature- and humidity-controlled colony room. The room was under a 12-h light/dark cycle (light onset 7.00 AM), and behavioral training and testing was conducted during the light phase (between 9.00 AM and 5.00 PM). Food and water was available ad libitum, but during training and probe test sessions, access to food was restricted (20–25 g/d) to maintain their body weight at around 90%–95% of free-feeding weight. The rats were handled for 5 days before the beginning of behavioral procedures. All experiments were approved by institutional veterinary officer and performed in accordance with the U.K. Home Office regulations of animal experimentation [Animals (Scientific Procedures) Act 1986].

### 2.2 Experimental design

**Fig. 1:**
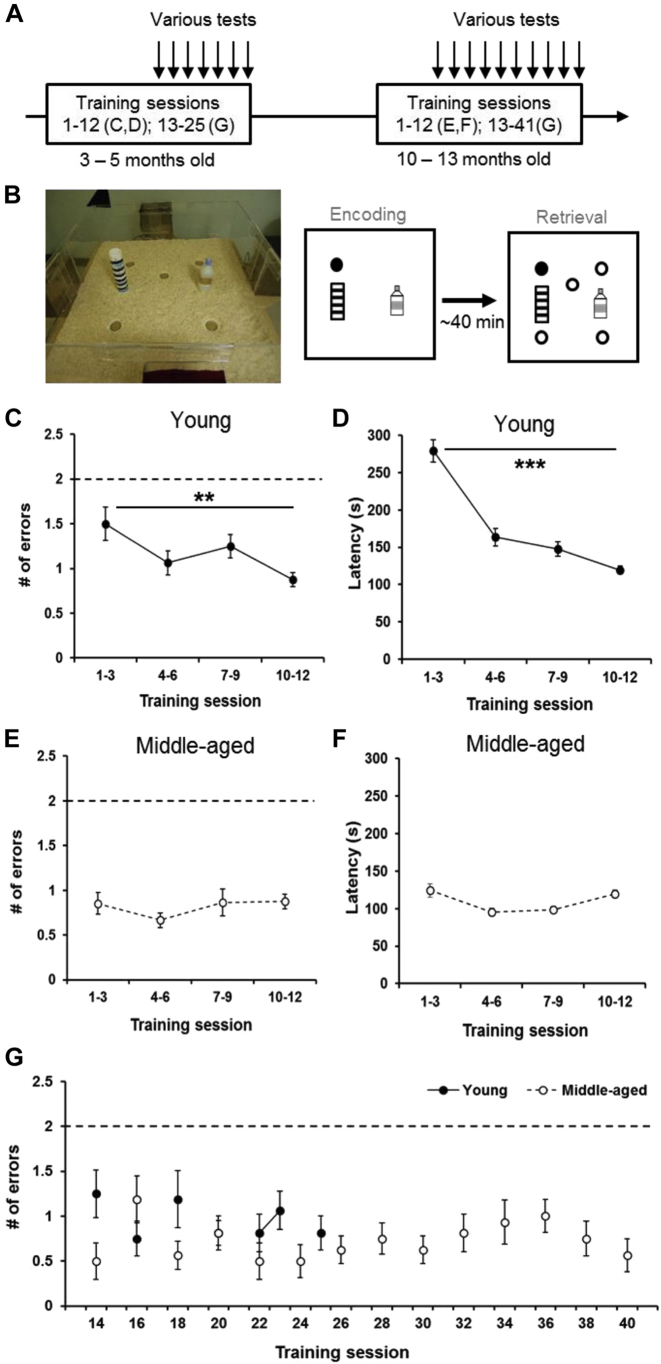
Training performance in the appetitive spatial task. (A) Experimental timeline. Rats were trained for 12 sessions and tested in various probe tests at 2 age ranges: 3–5 months old and 10–13 months old. (B) The event arena (left) and training paradigm (right). A training session was composed of 2 trials. During the encoding trial, the rat found hidden food rewards in a sandwell (filled circle) inside the arena. After a delay and during the retrieval trial, the rats could find more rewards in the location matching the encoding one (filled circle) but not in 4 other nonrewarded sandwells (open circle). (C) In young rats, the number of errors made during the retrieval trial gradually declined across the 4 blocks (average of 3 sessions per block) of training sessions. The dashed line indicates the chance level. (D) In young rats, the latency to retrieve the 3 pellets during the retrieval trial linearly declined across the 4 blocks of training sessions. (E) In middle-aged rats, the number of errors made at the retrieval trial was stable across the 4 blocks of training and was significantly below the chance level. (F) In middle-aged rats, latency to retrieve rewards at the retrieval trial was stable across the 4 blocks of training. (G) Errors in collecting the reward at the retrieval trial during interleaved training were below chance. All data are presented as mean ± SEM. ** *p* < 0.01, *** *p* < 0.001.

The same cohort of rats was trained and tested at 2 age stages for this longitudinal study. All rats were first trained and tested at the age of 3–5 months and again at the age of 10–13 months (Fig. 1A). At each stage, they received 12 training sessions followed by various encoding and probe tests with interleaving training sessions to evaluate their memory persistence.

### 2.3 Apparatus for the appetitive spatial memory task

All experiments were conducted in an event arena (135 × 135 × 40 cm, made of clear Plexiglas walls and white Plexiglas floor, Fig. 1B) lined with ∼2-cm sawdust and containing 2 intramaze landmarks. There were 4 start boxes (30 × 25 × 30 cm), placed in the center of each wall, covered with red filter paper that darkened the box and equipped with automated doors under the control of the experimenter. Chocolate-flavored food pellets (0.5 g per pellet, Supreme Mini Treats, ref: F05472, Bio-Serv) were used as rewards in this task. Plexiglas sandwells (6-cm diameter, 4-cm depth) could be inserted into the floor of the arena at different locations. To mask olfactory cues emanating from the reward, these sandwells were filled with mixture consisting of 95% bird sand and 5% ground food pellets. In addition, at the bottom of every sandwell, 4 g of food pellets was kept out of reach of the animals by a metal mesh divider to minimize the possibility of using olfactory cues to identify the rewarded sandwell. The arena was placed in a rectangular laboratory room with extramaze visual cues.

### 2.4 Box for novelty exploration

A square (100 × 100 cm) Plexiglas box with opaque white walls was used. To introduce novelty, we arranged different substances and textures, such as aquarium small pebbles, polished stones, plastic sealing clips, and small pieces of wood cuts, on a white Plexiglas floor or a floor that was lined with a yellow plastic sheet. Rats are very sensitive to their environment and the substance on which they walk (Diamond, 2010).

### 2.5 Behavioral procedures

We first habituated animals to the apparatus and procedures and then trained them to perform the spatial memory task in the event arena. We then examined the memory persistence in various probe tests with different encoding strength, time delay after encoding, and with or without MMEs after encoding.

#### 2.5.1 Habituation

Rats were handled every day for 5 days to habituate them to the experimenter and reduce their stress level. They were weighed daily to establish the baseline rate of weight gain under normal feeding and later underwent food restriction, as described previously, during habituation, training, and testing. There were 2 days of habituation to digging inside the sandwells and eating the reward. Sandwells with chocolate-flavored pellets were placed in their home cage where they naturally dug through the sand and obtained the pellets. They were then habituated to digging inside the sandwells in the event arena. First, the rats explored a quarter of the event arena (divided by removable walls) with a sandwell containing 4 pellets (1 pellet on top and 3 pellets in the middle of the sandwell). Second, the rats explored half of the event arena with a sandwell containing 4 pellets (1 pellet on top and 3 pellets in the middle of the sandwell). Third, the rats explored the whole event arena with a sandwell placed at the center of the arena containing 4 pellets (1 pellet on top and 3 pellets in the middle of the sandwell). During these habituation sessions, the animals explored the arena freely, found the food reward, and carried it to the start box to eat. The trial stopped when they found and ate all the 4 pellets.

#### 2.5.2 Training

Rats were trained in the event arena for 12 days at 5–6 days per week. A daily training session consisted of a memory encoding trial following by a retrieval trial 40 minutes later (Fig. 1B). During the encoding trial, 1 rewarded sandwell was placed in the arena at a particular location and provided an opportunity for each rat to encode where the food was available that day. The rewarded location (e.g., far from or near the start box) was counterbalanced across all rats to avoid bias toward certain part of the arena. Rats were given a single 0.5-g pellet in the start box before the door opened to accustom the animals to eat at the start box. After the door opened, rats explored the arena, found the sandwell location, dug to find the hidden reward, and then carried the pellet back to the start box to eat. The rats repeated these procedures until they collected the 3 pellets. The rewarded sandwell location and the start box location (north, east, south, and west) would change across days to encourage the animal to encode a new location on different days. The retrieval trial was a choice trial in which 5 different sandwell locations were present, but only the same sandwell location that matched the encoding trial would contain the rewards. If the rewarded location during the encoding trial was remembered, the animal would return to the matching location to find more rewards. The trial ended after the rats had retrieved and eaten the 3 pellets.

#### 2.5.3 Probe tests

After the initial 12 sessions of training, rats received various encoding-probe test conditions that were interleaved with regular rewarded encoding/retrieval training sessions. A typical condition consisted of an encoding trial with a rewarded sandwell, followed by a probe trial with 5 nonrewarded sandwells, as previously described (Salvetti et al., 2014, Wang et al., 2010). The probe trial was 60-seconds long with 1 of the 5 sandwells placed at the matching location to the encoding trial. After 60 seconds, the experimenter placed 1 pellet at the surface and 2 pellets at the bottom of the matching sandwell, so rats could find, retrieve, and eat the pellets. This was to avoid a weakening of the ability to use the matching principle to search due to nonrewarded probe tests. Some conditions were designed to evaluate if novelty after encoding facilitated memory persistence. In this case, rats received the encoding trial, returned to their home cage, and 30–40 minutes later were placed in the novel box for 5 minutes of exploration. Counterbalancing between paired conditions (e.g., with and without novelty) was carefully carried out, described in the results, and summarized in Table 1.

**Table 1:** Chronological order of training and probe tests in different conditions

Order	Experimental condition	Probe test	Age	Figure
1	Training		3–4 mo	1C,D,G
2	Short-term retention of 1- or 3-pellet encoding	1, 2	4 mo	2B–C
3	Long-term retention of 3-pellet encoding, or 1-pellet encoding with or without novelty	3, 4, 5	4 mo	3C, 4B
4	Long-term retention after reactivation with or without novelty	6, 7	5 mo	5A,B
5	Training		10–11 mo	1E–G
6	Long-term and intermediate-term retention of 3-pellet encoding	1, 2	11 mo	4C
7	Intermediate-term retention of 1-pellet encoding with or without novelty	3, 4	12 mo	3E
8	Long-term retention of 1-pellet encoding with or without novelty	5, 6	12 mo	3D
9	Short-term retention of 1-pellet encoding	7	12 mo	2D
10	Long-term retention of 3-pellet encoding with a second trial, novelty, or encoded zone	8, 9, 10	13 mo	4F–H
11	Long-term retention after reactivation with or without novelty or after novelty without reactivation	11, 12, 13	13 mo	5C–E

### 2.6 Behavioral analysis

During training, the accuracy of memory retrieval was measured by the number(s) of nonmatching (i.e., wrong) sandwells dug by each rat before they dug in the correct sandwell during the retrieval trial. The efficiency of retrieval was measured by the latency (in seconds) to find pellets in the rewarded sandwell during the retrieval trial. In both cases, the learning curve was typically characterized by the reduction of errors and the latency across training sessions. For memory performance at probe tests, the time that rats spent digging (contact of the forepaws with the sandwell) in different sandwells was recorded for the first 60 seconds of the trial. Sniffing or touching the sandwell with the nose was not included. The percentage of time digging at the correct (i.e., matching to encoding) location over the total digging time constituted the correct digging %. The average of percentage of time digging at the nonmatching location over the total digging time constituted the wrong digging %. A custom-built LabView timer was used to record the digging time and latency. All measurements were taken by the experimenters who were not aware of the conditions to which the animals were assigned.

### 2.7 Statistical analysis

#### 2.7.1 Training analysis

Data were presented as 4 blocks of 3 training sessions for young and middle-aged rats. Performance in every 3 training sessions was averaged for each animal. The group average was presented as mean ± standard error of the mean (SEM) for each block. The number of errors was analyzed using repeated-measures 1-way analysis of variance across blocks followed by 2-tailed 1 sample t-tests to compare each block with the chance level. The chance level of errors was 2, and a score of 0 would mean that the animal dug at the correct location before other locations. The latency to obtain all rewards was analyzed using repeated-measures 1-way analysis of variance across blocks. The performance at the last block, when the animal was young, was compared to the performance at the first block when the animal was middle aged.

#### 2.7.2 Test analysis

All data were averaged across animals within each experimental condition and were presented as mean ± SEM. The percentage of digging in the correct sandwell location was compared with the mean percentage of digging in wrong sandwells using 2-tailed paired t-tests. The chance level for 5 sandwells was 20 %. The percentage of correct digging was compared between 2 different conditions using a 2-tailed paired *t*-test. Parametric tests were used as the data conformed to a normal distribution (Shapiro-Wilk test). For all statistical tests, the size of the population was n = 16. Statistical significance was set at *p* < 0.05. All statistical analysis was done using SPSS Statistics 22 (IBM).

## 3 Results

We investigated whether aging would affect the persistence of memory using the appetitive spatial task previously described (Bast et al., 2005, Nonaka et al., 2017, Salvetti et al., 2014, Wang et al., 2010). The same cohort of male Lister hooded rats (n = 16) was trained in an event arena, and then their memory was assessed in different conditions of tests at the age of 3–5 months and then at the age of 10–13 months (Fig. 1A). The different test conditions used are summarized in Table 1.

### 3.1 Training performance in young and middle-aged rats

In young rats, the number of errors decreased over the 4 blocks of training sessions, indicating that they acquired the principle of finding more rewards during the retrieval trial in the location matching to the encoding one (Fig. 1C, linear trend of reduction from 1.5 ± 0.19 errors in block 1 to 0.88 ± 0.08 error in block 4, F_1, 15_ = 8.7, *p* = 0.01). Moreover, the number of errors they made was significantly below chance in the last block (t_15_ = −14.1, *p* < 0.0001). The latency to retrieve the 3 pellets also showed a significant linear trend of decrease, suggesting that the rats became more efficient at performing this task (Fig. 1D, 279.2 ± 15 seconds in block 1 to 119.5 ± 5.1 seconds in block 4, linear effect F_1, 15_ = 85.19, *p* < 0.0001).

In middle-aged rats, the number of errors in the first block remained low (Fig. 1E, around 0.8 error per trial) and was similar to the number of errors made at the last training block when they were young (t_15_ = 0.17, *p* = 0.87). All training blocks were significantly below the chance level (t_15_ = −16.48 to −7.51, all *p* < 0.0001). The latency to retrieve the 3 pellets stayed stable from the beginning to the end of the training (Fig. 1F, F_1, 15_ = 0.142, *p* = 0.71). Moreover, the latency in the first training block for middle-aged rats was similar to the latency in the last training block when they were young (t_15_ = −0.12, *p* = 0.9). These results indicate that rats learned the task and improved their accuracy and efficiency at finding the rewards in the retrieval trials when they were young, and, more importantly, that they remembered the matching-to-place principle of the task after a period of 5 months without training. After 12 sessions of training, various probe tests were introduced with interleaving training sessions. The performances during interleaved retrieval trials showed that the number of errors made by the rats to find the correct sandwell remained significantly below chance (Fig. 1G, young, t_15_ = −9.05 to −28.25, *p* < 0.0001; middle aged, t_15_ = −7.5 to −19.87, *p* < 0.0001).

In this within-subject study, rats received training when they were young and when they were middle aged, which enabled determination of change in motivation and motor function in the same group of animals. The latency for them to retrieve all 3 pellets during late training in young and early (re-)training in middle age was comparable, indicating that their motivation to find food reward and motor function in navigating in the arena were not affected in the middle age (Fig. 1D and F, t_15_ = −0.12, *p* = 0.9). These results are consistent with previous studies that showed no decline in sensorimotor function in the middle age (Cès et al., 2018, Fouquet et al., 2011, Frick et al., 1995, Gage et al., 1989).

### 3.2 Short-term retention of the appetitive spatial memory is intact in middle age

**Fig. 2:**
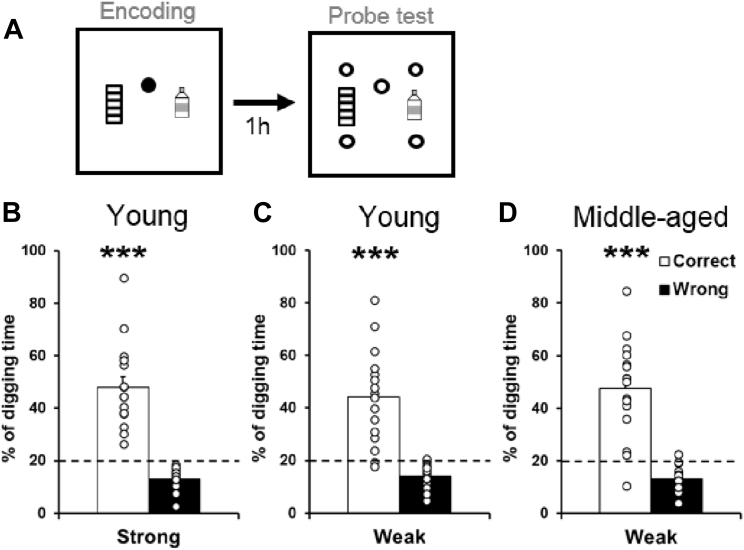
Short-term retention of appetitive spatial memory. (A) Behavioral procedures for short-term probe tests. Rats received weak (1 pellet) or strong (3 pellets) encoding (filled circle). One hour later, they were tested in a probe trial with 5 nonrewarded sandwells (open circles). (B–D) The percentage of correct digging was significantly higher than chance (dashed line) after strong (B) or weak (C) encoding in young rats and after weak encoding in middle-aged rats (D). Data are presented as mean ± SEM. *** *p* < 0.001.

We evaluated the short-term memory retention at 1 hour, after weak encoding with 1 pellet or strong encoding with 3 pellets (Fig. 2A). Young rats showed a significantly higher percentage of digging in the correct sandwell than chance, (Fig. 2B, 47.73 ± 4.05 %, t_15_ = 6.84, *p* < 0.0001; and Fig. 2C, 44.1 ± 4.5 %, t_15_ = 5.35, *p* < 0.0001), indicating that young rats retained the spatial memory at 1 hour, similar to what we have seen before (Wang et al., 2010). Middle-aged rats showed a significantly higher percentage of digging in the correct sandwell than chance as well (Fig. 2D, 47.5 ± 4.7 %, t_15_ = 5.9, *p* < 0.0001), and this was not significantly different from when they were young (cf. Fig. 2C, t_15_ = −0.44, *p* = 0.66). These results show that aging up to 10–13 months did not impair the short-term retention of a weak memory in this task.

### 3.3 Long-term retention of weak encoding is impaired by aging and is not facilitated by novelty in middle age

**Fig. 3:**
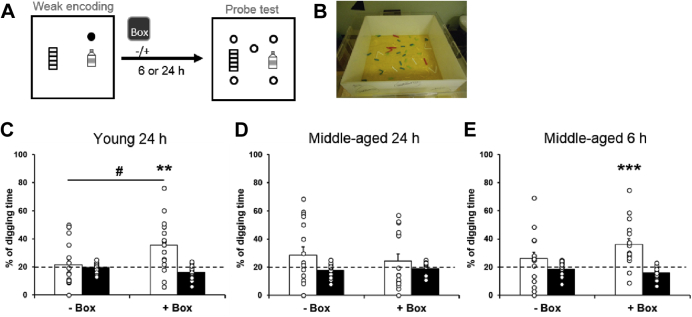
Long-term retention of weak appetitive spatial memory. (A) Behavioral procedures for long-term probe tests. Rats received weak encoding (filled circle). Twenty-four or 6 hours later, they were tested in a probe trial with 5 nonrewarded sandwells (open circles). Exploration in a novel box (gray square) was conducted or omitted 30–40 minutes after encoding. (B) An example of a novel box. (C) In young rats, the percentage of correct digging was not different from chance (dashed line) at 24 hours without novelty and was significantly above chance with novelty. (D) In middle-aged rats, the percentage of correct digging was not significantly different from chance at 24 hours, and novelty did not improve the percentage of correct digging. (E) In middle-aged rats, the percentage of correct digging was not significantly different from chance at 6 hours. With novelty, the percentage of correct digging was significantly higher than chance. Data are presented as mean ± SEM. ^#^*p* < 0.05, ** *p* < 0.005, *** *p* < 0.001.

We then investigated the long-term retention of weak encoding and the effect of exploration in a novel box on its persistence in young and middle-aged rats. Rats were given a weak encoding trial (Fig. 3A) with or without (in counterbalanced order) exploration in a novel box (Fig. 3B) 30–40 minutes after encoding (Fig. 3B). Without novelty, young rats did not show a significantly higher percentage of digging in the correct sandwell than chance at 24 hours (Fig. 3C left, 21.62 ± 3.83 %, t_15_ = 0.42, *p* = 0.68). With novelty, the correct digging percentage was higher than chance (Fig. 3C right, 35.4 ± 4.6 %, t_15_ = 3.35, *p* = 0.004) and also higher than the encoding without novelty (t_15_ = 2.6, *p* = 0.02). In middle-aged rats, the percentage of digging in the correct sandwell was not higher than chance at 24 hours in either condition (without box 28.5 ± 5.9 %, t_15_ = 1.45, *p* = 0.17; with box 24.4 ± 5 %, t_15_ = 0.87, *p* = 0.39), and the difference between conditions was not significant (t_15_ = −0.6, *p* = 0.56).

We tested memory retention after an intermediate delay (6 hours) in middle-aged rats (Fig. 3E). When strong encoding was used, the percentage of correct digging was significantly above chance at the 6-hour test (data not shown, 47.3 ± 4.6 %, t_15_ = 5.9, *p* < 0.0001). When weak encoding was used without novelty, middle-aged rats did not show a significantly higher percentage of digging in the correct sandwell than chance at 6 hours (Fig. 3E left, 26.1 ± 4.5 %, t_15_ = 1.33, *p* = 0.2). When novelty was introduced after weak encoding, the correct digging percentage became significantly higher than chance (Fig. 3E right, 36.1 ± 4.1 %, t_15_ = 3.9, *p* = 0.001). The difference between the presence and absence of novelty was not significant at a 2-tailed test (Fig. 3E, t_15_ = 1.83, *p* = 0.09) but was significant at a 1-tailed test (i.e., with > without novelty, *p* = 0.046). These results suggest that novelty could facilitate memory persistence of weak encoding in young but not in middle-aged rats.

### 3.4 Long-term retention of strong encoding is impaired by aging, and this can be reversed by strengthening the tagging process in middle age

**Fig. 4:**
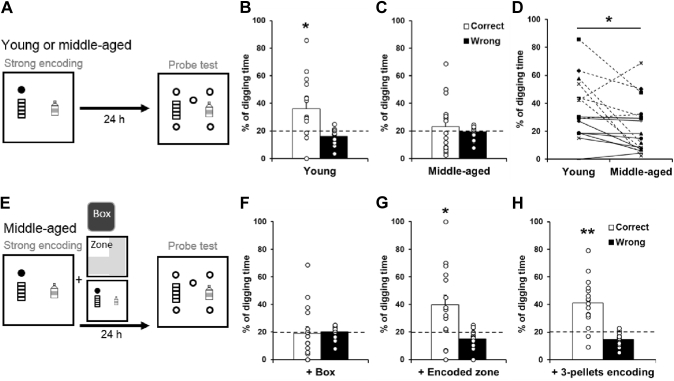
Long-term retention of strong appetitive spatial memory. (A) Behavioral procedures for long-term probe tests. Rats received strong encoding (filled circle). Twenty-four hours later, they were tested in a probe trial with 5 nonrewarded sandwells (open circles). Exploration in a novel box (gray square) was conducted or omitted 30–40 minutes after encoding. (B) In young rats, the percentage of correct digging was significantly higher than chance (dashed line). (C) In middle-aged rats, the percentage of correct digging was not different from chance. (D) Within-subject comparison between young (B) and middle-aged rats (C). (E) Middle-aged rats received strong encoding, and 3 different memory-modulating events took place 30–40 minutes later. Twenty-four hours after encoding, they were tested in a probe trial with 5 nonrewarded sandwells (open circles). (F) Novelty after encoding did not improve the percentage of correct digging, which was not different from chance. (G) Exploration in the encoded zone after encoding increased the percentage of correct digging, which was significantly higher than chance. (H) With a second strong encoding trial, the percentage of correct digging was significantly higher than chance. Data are presented as mean ± SEM. * *p* < 0.05, ** *p* < 0.005.

We investigated the 24-hour long-term retention of strong encoding (3 pellets) in young and middle-aged rats (Fig. 4A and E). Young rats showed a significantly higher percentage of digging in the correct sandwell compared with chance (Fig. 4B, 36.1 ± 5.4 %, t_15_ = 2.98, *p* = 0.009). In contrast, middle-aged rats did not show more digging in the correct sandwell compared with chance (Fig. 4C, 23 ± 4.8 %, t_15_ = 0.62, *p* = 0.55). The age effect was significant (Fig. 4D, t_15_ = −2.53, *p* = 0.02). This result indicates that aging impairs long-term retention of strongly encoded memory. Novelty after strong encoding (Fig. 4E) did not improve the long-term retention in middle-aged rats (Fig. 4F cf. Fig. 4C, t_15_ = −0.65, *p* = 0.53), and the correct digging percentage was not higher than chance (Fig. 4F, 19.1 ± 4.8 %, t_15_ = −0.19, *p* = 0.85).

To determine whether aging is primarily affecting the synaptic tagging process triggered by encoding or the PRPs produced by the novelty, we tested if a procedure to improve tagging, that was not confounded by reward-triggered PRPs, could improve memory persistence. For this, rats were returned to the encoded zone of the arena without rewards, after strong encoding. The percentage of correct digging was significantly higher than chance (Fig. 4G, 39.5 ± 7.2 %, t_15_ = 2.75, *p* = 0.016). Next, rats had another strong encoding trial 30–40 minutes after the initial encoding and showed a correct digging percentage that was higher than chance (Fig. 4D, 41.2 ± 4.4 %, t_15_ = 4.81, *p* = 0.002). Moreover, if middle-aged rats had a stronger encoding trial with 6 pellets, their correct digging percentage at 24 hours later was higher than chance (41.6 ± 6.4 %, t_15_ = 3.37, *p* = 0.004). Taken together, these results suggest that with aging, rats need stronger encoding or re-exposure to the encoding location to maintain the memory after a long delay.

### 3.5 Novelty improves memory persistence through memory reactivation and reconsolidation

**Fig. 5:**
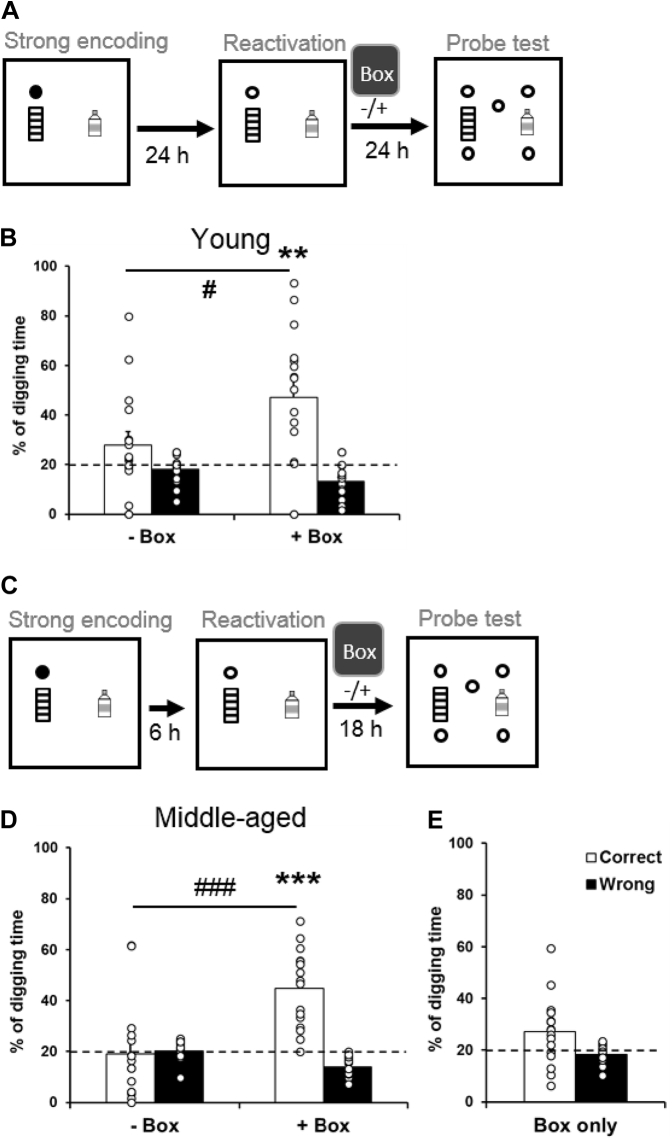
Memory retention after strong encoding and nonrewarded reactivation. (A) Behavioral procedures: young rats received a strong encoding trial (filled circle), a reactivation trial with a nonrewarded sandwell (open circle) 24 hours later, and a nonrewarded probe trial a further 24 hours later. Exploration in a novel box (gray square) was conducted or omitted 30–40 minutes after reactivation. (B) In young rats, the percentage of correct digging was not different from chance without novelty and was significantly above chance with novelty. (C) Behavioral procedures: middle-aged rats received a strong encoding trial (filled circle), a reactivation trial with a nonrewarded sandwell (open circle) 6 hours later, and a nonrewarded probe trial 18 hours later. Exploration in a novel box (gray square) was conducted or omitted 30–40 minutes after reactivation. (D) In middle-aged rats, the percentage of correct digging was not different from chance without novelty and was significantly above chance with novelty. (E) Exploration in a novel box at 6.5 hours after encoding without reactivation did not increase the percentage of correct digging compared to the reactivation only condition (D left). Data are presented as mean ± SEM. ^#^*p* < 0.05, ^###^*p* < 0.001, ** *p* < 0.005, *** *p* < 0.001.

Young rats received an encoding trial with 3 pellets followed by a nonrewarded trial 24 hours later to reactivate the memory that was, or was not, followed by exploration in a novel box 30–40 minutes afterward. They were then tested in a probe trial 24 hours after reactivation (Fig. 5A). Without novelty after reactivation, the percentage of correct digging was not different from chance (Fig. 5B left, 27.9 ± 5.4 %, t_15_ = 1.47, *p* = 0.16). With novelty, the percentage of correct digging was significantly higher than chance (Fig. 5B right, 47.2 ± 6.9 %, t_15_ = 3.9, *p* = 0.0013), and difference between conditions was significant (t_15_ = 2.17, *p* = 0.046).

To assess memory reconsolidation in middle-aged rats, shorter time windows between encoding and nonrewarded reactivation (6 hours) and between reactivation and the probe trial (18 hours) were used (Fig. 5C). Novelty was introduced, or omitted, 30–40 minutes after reactivation. Without novelty, the percentage of correct digging was not different from chance (Fig. 5D left, 19.1 ± 4.7 %, t_15_ = −0.19, *p* = 0.85). With novelty, the percentage of correct digging was significantly higher than chance (Fig. 5D right, 44.7 ± 3.8 %, t_15_ = 6.43, *p* < 0.0001), and the difference between conditions was significant (t_15_ = 4, *p* = 0.001). In middle-aged rats, novelty only, without reactivation, did not lead to a significantly higher correct digging percentage than chance (Fig. 5E, 27 ± 3.3 %, t_15_ = 2.12 *p* = 0.051) and was not significantly different from the condition of reactivation without novelty (t_15_ = 1.25 *p* = 0.23).

## 4 Discussion

Using this appetitive spatial paradigm, similar to daily experiences in humans, we found that short-term (1 hour) memory after weak encoding remained good, whereas long-term (24 hours) memory after strong encoding was already impaired at the middle age. In contrast to young rats, we found that novelty did not facilitate long-term memory persistence of weak or strong encoding in middle-aged rats. To differentiate whether it is the tagging process or the PRP production that is primarily impaired at the middle age, we found that a second strong encoding trial or a nonrewarded exposure in the encoded zone were able to improve long-term memory persistence. Moreover, introducing novelty after a nonrewarded memory reactivation facilitated memory persistence through reconsolidation.

### 4.1 Memory decline at middle and older ages

It has been widely shown that age-related memory decline is taking place in rodents aged beyond 20 months (Barnes et al., 1980, Creer et al., 2010, Gallagher and Burwell, 1989, Gallagher and Pelleymounter, 1988, Johnson et al., 2017). For example, spatial reference memory in the water maze is impaired in 24- to 32-month-old rats (Burke et al., 2012, Gage et al., 1984, Gallagher and Burwell, 1989, Holmes et al., 2010, Rapp et al., 1987) but is intact in 9- to 15-month-old rats and mice (Burke et al., 2012, Calhoun et al., 1998, Das and Magnusson, 2008, Magnusson, 1998, Magnusson, 2001). Spatial delayed-matching-to-place memory in the water maze is impaired in 22-month-old rats but stays intact at 12 months (Means and Kennard, 1991). In our appetitive spatial paradigm, we detected impairment of long-term memory retention in 10- to 13-month-old rats, suggesting that this paradigm is more sensitive for detecting decline in memory function. A key difference between the spatial tasks in the water maze and in the event arena is that the former behavior is driven by aversive experiences in the water. Therefore, it is possible that the spatial memory task in the water maze leads to a moderate level of stress (Harrison et al., 2009) and/or a potential metaplastic change (Abraham, 2008, Li et al., 2017) seen in amygdala (Karst et al., 2010) that contributes to memory consolidation (Roozendaal et al., 2009). The drive to escape from water is also likely to engage the brain networks responsible for emotion (Aguilar-Valles et al., 2006, Hadad-Ophir et al., 2014), and these functions are likely to preserve and contribute to emotional memories at a very old age (Denburg et al., 2003, Kensinger et al., 2002, Leal et al., 2017). Consistent with this view, studies have shown that fear learning and memory is intact in 24-month-old rodents (Bergado et al., 2011, Foster et al., 2012, Kennard and Woodruff-Pak, 2011, Oler and Markus, 1998, Wheelan et al., 2015).

### 4.2 Impaired tagging and capture mechanisms in middle age

The observed result of intact short-term memory may initially suggest that aging up to 10–13 months does not affect memory encoding. From the view of cellular consolidation (Frankland and Bontempi, 2005, McGaugh, 1966, Morris, 2006), the impaired long-term retention of memory can reflect impairment of protein synthesis important for memory consolidation, in particular the production of PRPs (Frey and Morris, 1997, Redondo and Morris, 2011, Schimanski and Barnes, 2010). However, our data suggest this is unlikely to be the primary mechanism that is affected in the middle age for 2 reasons. First, if spatial novelty-induced PRPs were significantly reduced in the middle age, spatial novelty would not enable memory persistence at 6 hours. Second, if the reduction in PRPs was causing this impairment in the middle age, then exploration in a familiar encoded zone should not improve memory persistence. This is because this procedure is unlikely to produce PRPs. Numerous studies have shown that exploration in a familiar environment does not enable facilitation of memory persistence (Ballarini et al., 2009, Moncada and Viola, 2007, Nomoto et al., 2016, Wang et al., 2010), presumably due to the lack of PRP upregulation. Hence, it is probable that exploration in a familiar encoded zone that is not rewarded does not upregulate PRPs. However, the result here shows improvement of memory persistence in middle-aged rats by exposure to the encoded zone after strong encoding. Exploration in the previously encoded zone provides rats with the opportunity to re-experience the surrounding where they encode the specific location, which can possibly re-engage the underlying tagging mechanism. This second wave of tagging could potentially capture the PRPs from the previous rewarded encoding trial and, combining with the first wave of tagging and PRP production during encoding, strengthen the physiological changes for long-term retention of the memory. Together, our results suggest that the tagging mechanism is starting to degrade in the middle age before the impairment of PRP production becomes prominent. We have summarized the potential activation of tags and PRPs, in the young and middle age, in different conditions in Table 2.

**Table 2:** Summary of hypothetical changes, inferred from the behavioral data, in the event-related production of tags and PRPs in different conditions in young (left) and in middle-aged (right) animals

Condition	Young animals			Middle-aged animals					
	1	2	3	1	2	3	4	5	6
MOI	Weak	Weak	Strong	Weak	Weak	Strong	Strong	Strong	Strong
MME	n.a.	Novelty	n.a.	n.a.	Novelty	n.a.	Novelty	Encoded zone	2nd trial
Tags	↑	↑	↑	—	—	—	—	↑	↑
PRPs	—	↑	↑	—	↑	↑	↑	↑	↑
LTM (24 h)	Poor	Good	Good	Poor	Poor	Poor	Poor	Good	Good

Key: LTM, long-term memory; MOI, memory of interest; Weak, weak encoding; Strong, strong encoding; MME, memory-modulating events; n.a., not applied; PRPs, plasticity-related products.

↑ increase from baseline of no events, — insignificant change from baseline.

It is conceivable that at a later stage of aging, the production of PRPs is also impaired. At the electrophysiological level, it has been shown that in 17- to 18-month-old hippocampal slices, early LTP induced by a weak tetanus in CA1 cannot be converted to late LTP by a separate strong tenanus, suggesting the “strong-converting-weak” effect is impaired by aging. It is proposed that in aged neural networks, there is a reduction of PRPs and that therefore, due to competition, PRPs are only used by the strongly stimulated synapses as they cannot not be captured by the weakly stimulated synapses (Sharma et al., 2015).

Because the sandwell and reward are not provided during the exposure to the encoded zone, explicit memory recall or relearning does not occur, whereas implicit memory reactivation or spatial re-encoding is likely to take place. Through compartmental and temporal analysis using fluorescent in situ hybridization (Chawla et al., 2004), it has been shown that exposure to the same context twice, with a 25-minute gap in between exposures, engages overlapping cell populations in the CA1 (Guzowski et al., 1999), CA2 (Wintzer et al., 2014), and CA3 (Vazdarjanova and Guzowski, 2004, Vazdarjanova et al., 2002) in the hippocampus. It is possible that going back to the zone where rewarded spatial encoding has taken place in our paradigm also allows overlapping sets of cells to be activated. As such, the STC occurs at the encoded zone and contributes toward the long-term persistence of the memory.

From the view of memory reactivation, it has been shown that navigation in the space where learning took place can engage the recall and reconsolidation of the learned memory. In the water maze paradigm, it has been shown that a probe trial in which rodents preferentially swim near the trained/target quadrant (or zone) can engage memory reconsolidation in a reference memory task in which the escaping platform is at a fixed location (Kida et al., 2009, Rossato et al., 2015; but see; Morris et al., 2006 and discussion in; Wang and Morris, 2010). In a delayed-matching-to-place task in the water maze in which the platform location has to be updated everyday, memory reactivation with a nonreinforced probe trial can make the memory labile and sensitive to interruption by protein synthesis inhibition in the hippocampus (Morris et al., 2006). Our paradigm shares the same learning principle of delayed-matching-to-place, and it is also dependent on the hippocampus (Bast et al., 2005, Wang et al., 2010). It is possible that returning to the encoded zone triggers memory reactivation that engages cells involved in the original encoding. Optogenetics has been used to label cells that are activated by a learning event and later reactivate those cells for memory expression (Liu et al., 2012, Ramirez et al., 2014). However, the technical limitation on the time window for labeling the cells involved in a specific event in this approach (i.e., off doxycycline for 2–3 days) makes it difficult to differentiate the cell populations involved in 2 events separated by a shorter gap, as in our paradigm.

The background context, where foreground learning occurs, can also trigger association between the learning event and the background context. In auditory fear conditioning, while an auditory stimulus is the foreground cue that is contiguous to the foot shock, the background context is often associated with the foot shock and can trigger conditioned fear responses (Frankland et al., 1997, Phillips and LeDoux, 1992, Wang et al., 2009). Hence, in our paradigm, it can be perceived that while the specific sandwell location is proximal to the rewards and is being encoded for later retrieval, the zones surrounding the sandwell also gain association with the rewards and form memory traces.

In behavioral tagging and capture experiments, memory persistence and the procedures to improve it follow the same principle seen in STC. One may suggest that the aging effect on memory we have seen here reflects a change in functional plasticity. We cannot entirely rule out the potential structural change in the middle age. However, previous literature would suggest that changes in structure tend to occur at a stage much later than 10 months old. For example, neuronal loss is seen in 25-month-old rats, reduction in dendritic branching is seen in 18-month-old rats, and reduction in the hippocampal volume is seen in 21-month-old mice (Bettio et al., 2017, Burke and Barnes, 2006, Shetty and Sajikumar, 2017).

## 5 Conclusion

Understanding how aging affects learning and memory is crucial for improving healthy aging, but memory impairments can be subtle in the middle age and would remain undetectable. The present study provides new evidence that appetitive spatial memory is already impaired at an early stage of aging and provides new thinking on targeting STC as an effective strategy to preserve memory retention. This behavioral task, which is similar to everyday experiences in humans, allowed us to detect early age-related memory impairments and provides a model to study the impact of healthy aging or pathological aging such as Alzheimer's disease.

## Disclosure statement

The authors declare no conflicts of interest.
